# Creating a Registry for Patients with Thoracic Outlet Syndrome

**DOI:** 10.3390/diagnostics7020036

**Published:** 2017-06-17

**Authors:** Misty D. Humphries

**Affiliations:** Division of Vascular and Endovascular Surgery, University of California Davis Health, 4860 Y Street, Suite 3400, Sacramento, CA 95817, USA; mdhumphries@ucdavis.edu

**Keywords:** Thoracic Outlet Syndrome (TOS), nTOS, aTOS, vTOS, Paget-Schroetter syndrome

## Abstract

The creation of any patient database requires substantial planning. In the case of thoracic outlet syndrome, which is a rare disease, the Society for Vascular Surgery has defined reporting standards to serve as an outline for the creation of a patient registry. Prior to undertaking this task, it is critical that designers understand the basics of registry planning and a priori establish plans for data collection and analysis.

## 1. Introduction

A registry is defined as a place where data, records, or laboratory samples are kept and made available for research [1]. Registries have become powerful tools to observe the course of disease, understand variation in treatment and outcomes, describe patterns of care, and examine factors that influence prognosis and quality of life. Registries are particularly useful in the case of rare diseases where the ability to conduct clinical trials is hindered by the rarity of the condition and the aggregation of treatment to specialty centers. Thoracic Outlet Syndrome (TOS) is an example of a rare disease where diagnosis can be extremely challenging, especially in the case of neurogenic TOS. Nuances of treatment and post-operative care are critical to successful outcomes, and there are clear patient and disease characteristics that influence prognosis in TOS. The establishment of a TOS registry allows for aggregation of data, not just at one institution, but across centers, with the goal of comparing treatment and guiding management. Registries have allowed for the development of outcomes standards in rare disease and establish what providers in real-world practice can achieve. The Agency for Healthcare Research and Quality has produced a summary which can provide guidance and answer questions when attempting to create a registry [2].

## 2. Registry Planning

The creation of a registry begins with defining the purpose of the registry. There are five main purposes for registries: (1) describing the natural history of disease; (2) determining clinical and/or cost-effectiveness; (3) assessing safety or harm; (4) measuring or improving quality of care; (5) public health surveillance and disease control. Multiple studies have demonstrated disparities between results in clinical trials and results in clinical practice [3,4]. Efficacy of treatment for well-defined patient populations in trials may not be generalizable to other populations or subgroups. Improvement in comparative effectiveness methodologies in observational research has increased interest in the investment in registries across many stakeholders. Both the Institute of Medicine (IOM) and the Federal Coordinating Council for Comparative Effectiveness Research have identified patient registries as a core component of comparative effectiveness data infrastructure [5,6]. Registries are also expected to play an important role in the Patient Centered Outcomes Research Institute (PCORI) for their ability to provide information in the “real-world” setting and measure quality of care. Quality-based registries are increasingly able to assess differences between providers and patient populations based on performance measures and identify disparities, demonstrate opportunities for improvement, and provide transparency through public reporting. Most registries are developed with more than one purpose in mind. Additionally, registries designed with an initial purpose may be modified over time to accommodate additional purposes of research, practice, or policy environment changes.

The second step in planning a registry is identifying stakeholders. Stakeholders can play an integral part in design and function of a registry. They are invaluable at ensuring the registry is meeting its key objectives over time. This is particularly important for registries that collect data over many years. Stakeholders include patients, clinicians, providers, product manufacturers, and payers. In rare disease registries, such as TOS, stakeholders may also include advocacy groups, public health agencies, and scientists. Stakeholders are typically classified as primary—those that create and fund the registry—or secondary—those that would benefit from knowledge of the data or would be impacted by the results. Different stakeholders will perceive and benefit from the registry in different ways. For physicians, the registry can provide insight into management of a disease in accordance with evidence-based guidelines [7]. For patients and patient advocacy groups, a registry can increase understanding of the natural history of a disease, contribute to the development of treatment guidelines, or facilitate research on treatment [7,8]. When multiple stakeholders are involved, clear policies should be in place regarding governance, data access, and the publication of data from registries. It is also important that communication with stakeholders is consistent to maintain their interest in success of the registry.

A key element in determining the feasibility of a new registry is funding, which is especially true for national registries. Local registries, where the scope is more limited, may be set up with less expense. Specific factors that determine the feasibility of a registry include the number of sites, number of patients, the scope of data to be collected, and the methods used to collect data. Acquisition of data from the electronic health record through computerized means is highly feasible, but manual extraction of data imbedded in free text fields requires a team of people, which may preclude the collection of large amounts of data. Creating electronic portals into which participants can easily enter their own data at the initial visit and at home for follow-up can improve collection, especially in cases where participants may see multiple clinicians in many different fields. In the case of TOS, patients may see a physical therapist, a pain management specialist, and a surgical specialist. Collection of data from each visit can be facilitated by allowing the registry participant to enter data at home through regular emailed updates. This also improves the likelihood that participants will answer honestly and not be swayed by a provider.

## 3. Scope

The scope of a registry is viewed in terms of the size, setting, duration, geography, and financing. The purpose and objectives of the registry frame the scope, although other factors, such as research interests and disease specific guidelines, will also shape it. The scope of a registry is also affected by the degree of uncertainty acceptable to primary stakeholders. The amount of uncertainty is determined by weighing the quantity, quality, and detail of the data collected against its considered importance and value. Here, we will focus on two key concepts in scope: the core data set and patient outcomes.

Each data element included in the core data set should address the central questions for which the registry was designed. These should be balanced with noncore variables, such as more descriptive or exploratory ones. Balance is required in the use of core and noncore variables and reduces attempts to accomplish too many goals. When there are excessive noncore data elements, collecting data becomes a significant burden. This ultimately outweighs the usefulness to clinical sites and prevents them from participating. Even when core variables are limited and appear relatively easy to collate, the reliability of some variables can be suspect. The Society for Vascular Surgery has put forth a formal reporting standards document for patients with TOS. This document provides examples of data collection variables and specifics into which variables should be considered core and noncore [9]. Within a TOS registry, for example, the exact use of medications and the variable nature of medication reporting can call into question the reliability of whether patients are using narcotics or illegal substances to control their pain. Finally, consideration of what data are readily available may determine what will remain part of the core data. Data that are consistent with general practice are typically much easier to collect that data that exceed usual practice. This is especially true for quality-of-life data. For patients with TOS, there are multiple data forms that can be used to assess quality of life. Not all institutions can collect data from the Quick-Dash, the SF-12, and a brief pain inventory. Although collection of data from each survey yields more information about the patients’ functionality and life, for many practices, quality-of-life data are not an essential element collected in a routine history or physical. In creating our own database, we could have labeled quality-of-life data as a noncore data element. This would allow more institutions to participate, but may limit conclusions that can be made regarding treatment. For this reason, we focus on collecting these patient centered outcomes in order to better refine treatment. Furthermore, because there is not a TOS-specific quality-of-life form, many institutions may use their own forms or select one form over another. In addition to establishment of core and noncore elements, patient outcomes in the order of greatest importance should be identified early in the concept phase of the registry. Defining which outcomes will be primary and secondary forces prioritization within the design of the registry.

## 4. Data Collection

Data collection is a fluid process that should be pilot tested, adjusted, and retested several times prior to the full implementation of a clinical registry. This process, although onerous, is imperative to the long-term success of the registry. In the case of our registry, testing took 16 months to ensure the ease of use. An initial case report form should be developed. This is a formatted list of data elements that can be presented in paper or electronic form and is the data structure of a clinical registry. The case report form is developed as the purpose and scope are delineated with principles regarding the core and noncore elements, and then it is modified numerous times as the registry is pilot tested. Once the case report form has been conceived, a data dictionary of definitions and parameters should be developed. The data dictionary should describe each data element and provide information about how the data should be interpreted. This is especially important for data which are to be extracted from a chart by research personnel who may not be as knowledgeable about the clinical condition.

The most successful registries utilize a collection system that can be integrated into day-to-day clinical practice. The case report should be broken down by the type of clinical visit; initial evaluation, surgical treatment, non-surgical treatment, and follow-up. Specific data elements tailored for each type of visit, as well as patient reported outcomes, are collected by translating the case report form into a two-part document for the clinician and the patient to complete. Although paper forms may be used, electronic versions of data entry can simplify collection for patients, especially if there are multiple forms. In the case of the Vascular Quality Initiative database, the vast amount of data requires specialized personnel to extract data. In our own TOS database, data are extracted from the electronic medical record through a specific TOS clinical template. The creation of a mobile tablet-based case report form can be used in the waiting room after patients have checked in for their visit. In cases where there are multiple data forms or surveys, the presence of a research assistant may be required to help guide the participant through the data collection process. To simplify data collection by the clinician, the case report form should be converted into an electronic health record clinical documentation template. This allows for easy incorporation into practice and extraction by registry personnel. The paper-based form can be given to the clinician for use in guiding the visit, and then translated into the clinic visit note. This too ensures ease of extracting data for research personnel.

## 5. Data Analysis

Not all registries are developed with a testable hypothesis in mind, and this is perfectly reasonable. Studies that emerge from registries may initially present descriptive work that has largely been unknown, such as clinical progression. In registries where the aim is to study the association between a specific exposure and outcome, prespecification of the study methodology and the establishment of a prior hypothesis may affect the acceptance of results derived from the registry. On the other hand, a study may evolve out of an unexpected observation in the database during analysis for other purposes, or may evolve from a concerted effort of the registry participants to answer a specific question. Regardless, transparency in methods is essential in order to allow the reader to understand whether the analysis evolved from multiple iterations of exploratory analysis or whether it was a hypothesis developed independently of the registry.

During any analysis of registry data, the first step is to assess the data quality. Missing data can represent a challenge for any registry-based analysis. Missing data can include situations where one question in a group of variables is not answered, such as a patient-reported survey. In this type of case, a decision must be made: should the entire patient record be removed? Or should parts of the data be analyzed as complete while other parts as missing? Removing the record completely reduces the information yield from the study, while analyzing partial records could seriously bias the results. One way to determine whether or not data are missing at random is to compare the distribution of observation variables for patients with specific missing data to the distribution of patients for whom that data are present. Though it may still be difficult to explain why the data are missing, this is an accepted analytical method for managing missing data.

While there are numerous methods of analyzing observational data, the decision to perform a descriptive or comparative analysis is the first step. Statistical methods used for descriptive purposes include summarization of continuous and categorical data, reporting incidence and prevalence of a disease, and incidence rate. Descriptive studies that include follow-up can provide insight into the number of patients that are frequently lost to follow-up. In cases where patients provide data, information can be garnered about change in providers over time. When comparative analysis is performed from registry data, there are limitations to which associations can be drawn, and the importance of confounding must be considered. Although planning of the registry attempts to account for as many confounders as possible, the use of advanced statistical methods—such as stratified analysis, multivariable analysis, propensity scoring, or instrumental variable analysis—may be needed. It is also important in comparative analysis to consider the extent to which bias, especially detection and selection bias, can distort the results. Development of a statistical plan at the onset of analysis to address the primary and secondary objectives of the research question, as well as the overlying hypothesis, drastically simplifies the analytical process and has the best chance of producing a research product with results that can be generalized.

## 6. Summary

The use of registry data is only going to become more prolific as the cost of randomized control trials increases and patients with increased knowledge about treatment options refuse randomization. In today’s era of team science, more collaboration between institutions has allowed registries to be supported at one facility, yet still recognize other institutions and participants. While developing a registry is a huge undertaking, it is not necessary to reinvent the wheel. Well established registries in other areas can provide a base for data collection while giving you access to a structured reporting system and the possibility of collaboration. In our own TOS registry, we allow participation from any other institution. We support other institutions by providing support for patient data collection and supplying data case forms for collection. We also have established electronic portals for both patients and providers to enter data, and we even support entry of data for institutions that do not have resources to extract patient data and enter it into the system. This is all done at no cost to the institution other than support of a resource contact person.

## References

[1] Merriam-Webster Registry 2017. www.merriam-webster.com.

[2] Gliklich R., Dreyer N., Leavy M. (2014). Registries for Evaluating Patient Outcomes: A User’s Guide.

[3] MacIntyre K., Capewell S., Stewart S., Chalmers J.W., Boyd J., Finlayson A., Redpath A., Pell J.P., McMurray J.J. (2000). Evidence of improving prognosis in heart failure: Trends in case fatality in 66,547 patients hospitalized between 1986 and 1995. Circulation.

[4] Wennberg D.E., Lucas F.L., Birkmeyer J.D., Bredenberg C.E., Fisher E.S. (1998). Variation in carotid endarterectomy mortality in the Medicare population: trial hospitals, volume, and patient characteristics. JAMA.

[5] Institute of Medicine (US) Roundtable on Value & Science-Driven Health Care (2007). Learning What Works Best: The Nation’s Need for Evidence on Comparative Effectivenss in Health Care.

[6] Research FCCfCE (2009). Report to the President and the Congress. http://www.hhs.gov/recovery/programs/cer/cerannualrpt.pdf.

[7] LaBresh K.A., Gliklich R., Liljestrand J., Peto R., Ellrodt A.G. (2003). Using “get with the guidelines” to improve cardiovascular secondary prevention. Jt. Comm. J. Qual. Saf..

[8] Charrow J., Esplin J.A., Gribble T.J., Kaplan P., Kolodny E.H., Pastores G.M., Scott C.R., Wappner R.S., Weinreb N.J., Wisch J.S. (1998). Gaucher disease: Recommendations on diagnosis, evaluation, and monitoring. Arch. Intern. Med..

[9] Illig K.A., Donahue D., Duncan A., Freischlag J., Gelabert H., Johansen K., Jordan S., Sanders R., Thompson R. (2016). Reporting standards of the Society for Vascular Surgery for thoracic outlet syndrome. J. Vasc. Surg..

